# S‐acylation of Ca^2+^ transport proteins in cancer

**DOI:** 10.1002/cdt3.146

**Published:** 2024-08-14

**Authors:** Sana Kouba, Nicolas Demaurex

**Affiliations:** ^1^ Department of Cell Physiology and Metabolism Centre Médical Universitaire, University of Geneva Geneva Switzerland

**Keywords:** Ca^2+^ signaling, Ca^2+^ transport proteins, cancer, S‐acylation, S‐palmitoylation

## Abstract

Alterations in cellular calcium (Ca^2+^) signals have been causally associated with the development and progression of human cancers. Cellular Ca^2+^ signals are generated by channels, pumps, and exchangers that move Ca^2+^ ions across membranes and are decoded by effector proteins in the cytosol or in organelles. S‐acylation, the reversible addition of 16‐carbon fatty acids to proteins, modulates the activity of Ca^2+^ transporters by altering their affinity for lipids, and enzymes mediating this reversible post‐translational modification have also been linked to several types of cancers. Here, we compile studies reporting an association between Ca^2+^ transporters or S‐acylation enzymes with specific cancers, as well as studies reporting or predicting the S‐acylation of Ca^2+^ transporters. We then discuss the potential role of S‐acylation in the oncogenic potential of a subset of Ca^2+^ transport proteins involved in cancer.

## Key points

- S‐acylation is a reversible lipid post‐translational modification that adds a long‐chain fatty acid to specific cysteine residues of target proteins.
- S‐acylation is mediated by membrane zDHHC enzymes, also known as protein acyl transferases, and is reversed by de‐acylases or acyl protein thioesterases.
- Enzymes that mediate the addition and removal of lipids via S‐acylation are implicated in cancer.
- Calcium transporters are signaling proteins implicated in cancer growth and progression.
- Calcium transporters are targets for S‐acylation, and their lipid modification is relevant for cancer research.

## Graphical abstract

The implication of Ca^2+^ transport proteins in cancer has been previously documented in several studies recently reviewed. Transporters highlighted in red have been biochemically documented to be S‐acylated.

## 1 INTRODUCTION

Protein S‐acylation, also referred to as S‐palmitoylation, is a critical post‐translational modification that involves the reversible attachment of a long‐chain fatty acid, typically 16‐carbon palmitate acid, to specific cysteine residues of target proteins via a thioester bond.^1^ S‐acylation enhances protein hydrophobicity, promoting their interaction with non‐polar structures like lipid bilayers, thereby influencing protein subcellular localization, trafficking, and interactions with other molecules.^2^ This process is catalyzed by palmitoyl‐transferases proteins encoded by 23 genes in humans. Despite sharing a conserved Zinc finger Asp‐HiS‐HiS‐Cys (zDHHC) motif, these proteins exhibit diverse cellular distributions and unique substrate preferences and efficiencies during the acylation process. The removal of fatty acid groups is mediated by Acyl Protein Thioesterases (APT1&2) and α/β‐hydrolase Domain‐Containing Proteins (ABHD).^3^ The dynamic and reversible process of S‐acylation enables precise regulation of protein function in both space and time.

Cancer cells exhibit hallmark traits such as sustained proliferation, resistance to apoptosis, and increased metastatic potential due to abnormalities in intracellular signaling, metabolic pathways, and gene regulation networks.^4^ Several signaling proteins implicated in cancer are targets for S‐acylation^5^ including Ras proteins,^6^ Src family kinases,^7^ Wnt signaling components,^8^ Protein kinases C (PKC),^9^ and calcium (Ca^2+^) transporters.^10^ Abnormal Ca^2+^ signals contribute to carcinogenesis^11^ and post‐translational modifications of Ca^2+^ transporters have been linked to cancer,^12^ but the contribution of S‐acylation in this process remains unexplored.

In this review, we summarize recent work reporting the involvement of acylation enzymes in cancer, as well as studies documenting or predicting the S‐acylation of Ca^2+^ transporters. We then examine the potential impact of S‐acylation on the pathophysiology of a subset of Ca^2+^ transport proteins implicated in cancer.

## 2 S‐ACYLATION: KEY ENZYMES AND BIOCHEMICAL MECHANISMS

**Figure 1:**
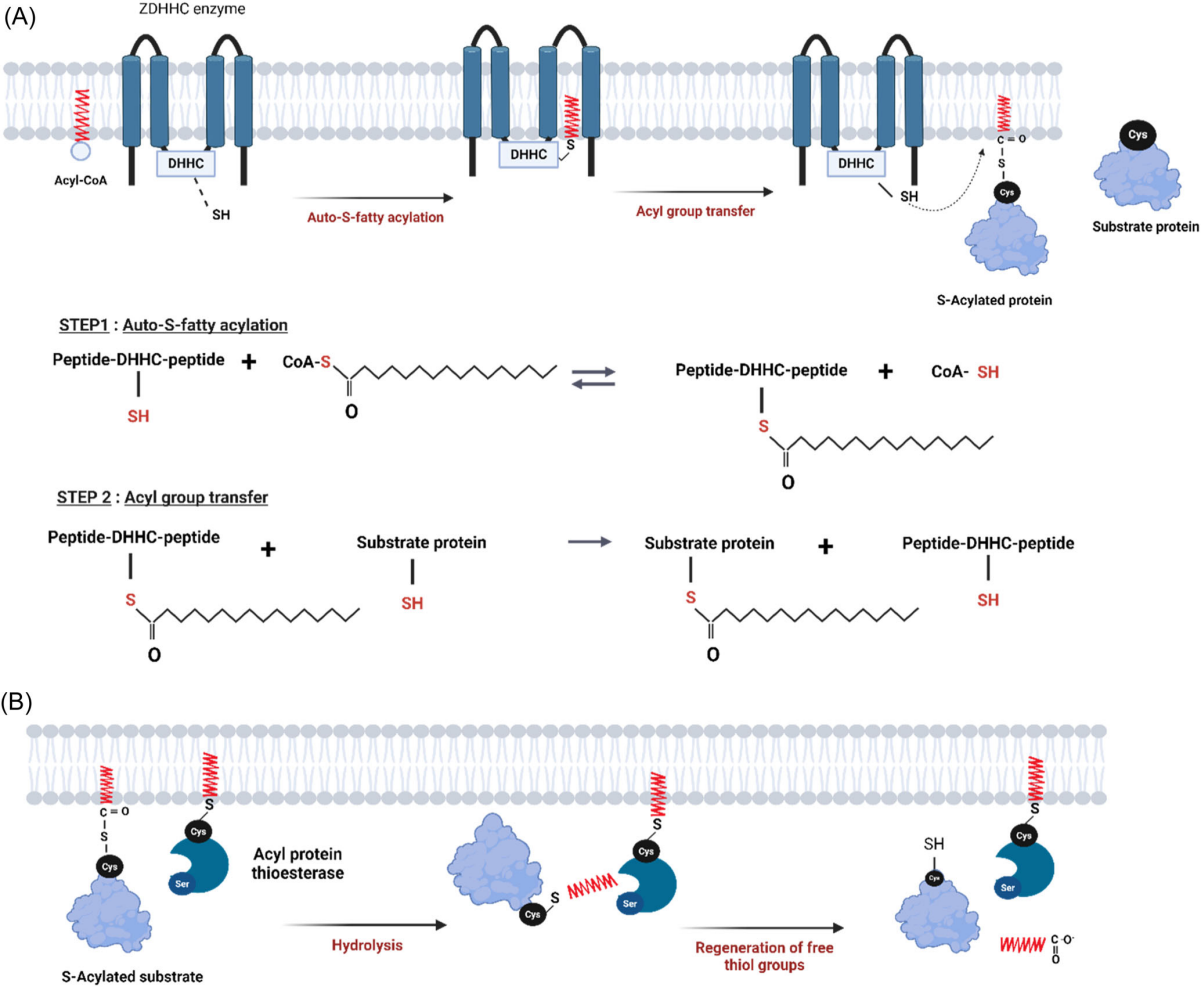
S‐acylation cycle and enzymes. (A) A target protein is S‐acylated at the thiol group of particular cysteine residue by zDHHC enzymes, using acyl‐CoA as acyl donor. zDHHC proteins are initially auto‐S‐fatty acylated on the DHHC cysteine residue, releasing free CoA, followed by the transfer of the acyl group to the acceptor cysteine residue of a substrate protein. (B) The thioester‐tethered lipidation is eliminated through hydrolysis mediated by deacylases such as APTs. APTs are themselves S‐acylated and contain a hydrophobic pocket to accept acylated substrates, positioning the lipidated cysteine of the substrate near the serine (Ser) residue of the active site. This process allows the regeneration of free thiol groups. zDHHC, Zinc finger Asp‐HiS‐HiS‐Cys. The figure was created with www.biorender.com.

The human genome encodes 23 zDHHC enzymes bearing four or six transmembrane (TM) domains, with the DHHC cysteine‐rich domain situated between TM 2 and 3. The catalytic reaction of zDHHC enzymes occurs through two sequential steps.^13^ First, the DHHC cysteine within the active site undergoes auto‐acylation by interacting with an acyl‐coenzyme A (acyl‐CoA) donor. Second, this acyl chain is transferred from the DHHC cysteine to cysteine residues of target proteins (Figure 1A). The majority of TM zDHHCs are situated in the endoplasmic reticulum (ER), while others are found in the Golgi, the nuclear envelope^14^ or dispersed throughout the early secretory pathway (ER‐Golgi intermediate compartment ERGIC pathway).^15^ Some zDHHCs exhibit dual localization^16^ at the plasma membrane (PM) and in the endocytic network.

De‐acylation enzymes employ a conserved catalytic serine to trigger a nucleophilic attack on the thioester carbonyl group,^17^ producing an intermediate ester subsequently hydrolyzed by water. De‐acylation enzymes comprise acyl‐protein thioesterases (APT1 and APT2, encoded by the *LYPLA1* and *LYPLA2* genes^18^), palmitoyl‐protein thioesterase 1 and 2 (PPT1 and PPT2),^19^ and ABHD.^20^, ^21^ De‐acylation begins with acyl‐thioesterases binding to cell membranes (Figure 1B), via electrostatic interactions and the insertion of a hydrophobic loop into the membrane.^17^ S‐acylation anchors thioesterases to the membrane, enabling the enzymes to detach the acyl chain from the membrane. They then position it within a hydrophobic pocket to ensure proper engagement of the thioester bond at the active site. Thioesterases exhibit diverse cellular distribution, with PPT1 and PPT2 localized inside late endosomes and/or lysosomes,^3^ APT2 and some ABHDs in the Golgi and PM, and others trafficked to different cellular compartments.^17^ How thioesterases navigate throughout the endomembrane systems to interact with their substrates is unclear, but their labile interactions with membranes enable a dynamic regulation of protein S‐acylation.^22^

## 3 TECHNICAL ASSAYS TO STUDY PROTEIN S‐ACYLATION: BENEFITS AND LIMITATIONS

### 3.1 Metabolic labeling with radiolabelled palmitate

Radiolabelling techniques were developed in the 1980^23^ with the aim of tagging cellular fatty acid pools using tritiated palmitate ([3H]‐palmitate), followed by purification, electrophoresis, and autoradiography.^24^ Despite their effectiveness in revealing rapid S‐acylation dynamics, the use of [3H]‐palmitate has declined due to challenges associated with significant sample amounts and long exposure times for autoradiographs. It is also limited in providing insights into S‐acylation stoichiometry. There are also other limitations for radiolabelling, such as the preference of some zDHHC‐PATs for fatty acid chain lengths other than 16 carbons and the potential metabolism of labeled palmitate, leading to its incorporation into different cellular fatty acid pools.

### 3.2 Acyl‐biotin exchange and acyl‐resin assisted capture

**Figure 2:**
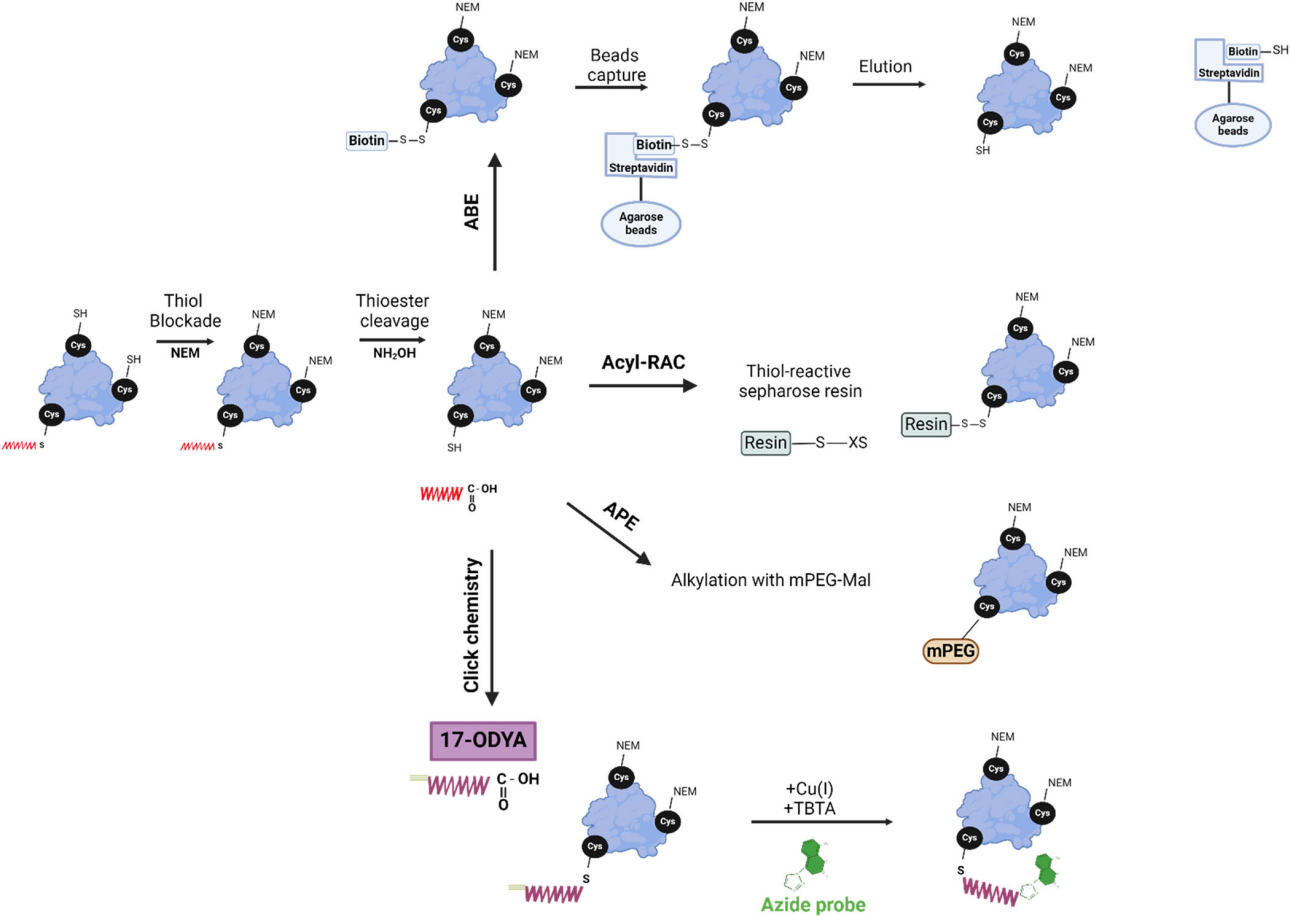
Methods to study S‐acylation. Acyl‐biotin exchange (ABE): Cysteines are blocked with NEM. Thioesters are then cleaved using NH_2_OH, and the resulting cysteines are reacted with HPDP‐biotin (pyridyldithiol‐biotin). Proteins are captured with streptavidin beads, eluted with reducing agents, and analyzed via Western blot or mass spectrometry. Acyl‐resin assisted capture (Acyl‐RAC): Following thiol blockade and NH_2_OH cleavage, newly generated cysteines are bound to thiopropyl sepharose‐containing beads. By comparing the unfractionated lysate with the captured proteins, the level of S‐acylation can be determined via Western blot or mass spectrometry. Acyl‐peg exchange (APE): After NH2OH cleavage, newly generated cysteines are reacted with 5 kDa methoxy‐PEG‐maleimide (mPEG‐Mal). Proteins labeled with mPEG‐Mal migrate slower on the gel. Proteins with different numbers of mPEG‐Mal are separated on the Western blot, allowing to identify the number of S‐acylation sites. Click‐chemistry: Based on the S‐acylation machinery of cells, an alkyne/azide‐containing fatty acid (commonly 17‐ODYA) is conjugated, followed by incubation with an azide‐containing click chemistry probe attached to a fluophore which can be analyzed by western blot or fluorescence microscopy and can be combined with mass spectrometry. The figure was created with www.biorender.com.

The acyl‐biotin exchange (ABE) assay^25^ enables affinity purification and proteome‐level characterization of S‐acylated proteins by converting the acyl modification into a stable biotin adduct (Figure 2). This process relies on the high reactivity of the thioester bond and its removal by weak bases such as hydroxylamine (NH_2_OH). Another assay, known as acyl‐resin‐assisted capture (Acyl‐RAC), improved the process by combining steps of thioester cleavage and capture by thiol‐reactive sepharose beads (Figure 2).^26^ Both Acyl‐RAC and ABE allow direct assessment of S‐acylation levels at steady state but they cannot capture S‐acylation/de‐acylation dynamics or changes in S‐acylation states. They also require multiple washing steps after cysteine alkylation and/or biotinylation. To ensure complete alkylation of free cysteines and specificity in thioester cleavage, strategies such as initial reducing steps are employed to help reduce false positives.

### 3.3 Cysteine site‐specific PEGylation

This technique is an adaptation of the ABE/Acyl‐RAC assays (Figure 2). After thioester cleavage, the resulting free thiol is capped with PEG‐N‐ethylmaleimide to introduce a mass shift compared to the non‐acylated protein. This causes a “laddering” effect in the protein of interest on sodium dodecyl sulphate‐polyacrylamide gel electrophoresis (SDS‐PAGE) and western blot.^27^ The degree of laddering reveals the number of S‐acylation sites and the stoichiometry of S‐acylation. Available cysteine‐reactive PEG maleimides, induce a 5 kDa or 10 kDa shift. However, this technique is limited to proteins migrating as single bands smaller than 100 kDa on SDS‐PAGE. Nevertheless, PEGylation remains the only reliable method for quantifying S‐acylation sites and detecting transitions between different S‐acylation states.

### 3.4 Click chemistry

Click chemistry is based on metabolic labeling of a clickable analog of palmitic acid, typically 17‐octadecyonic acid (17‐ODYA). This step is followed by copper‐catalyzed alkyne‐azide cycloaddition reaction (Cu(I)‐catalyzed [3 + 2] Huisgen) known as “Clicking” and incubation with an appropriate azide or alkyne‐containing click chemistry probe^28^ (Figure 2). These probes have been helpful in identifying new acylated proteins, validation of modification sites, and understanding of fatty acid selectivity, including the characterization and manipulation of carbon lengths and zDHHC‐PAT preferences. This technique is limited by low stoichiometry protein detection but reduces false positives compared to ABE/AcylRAC.

## 4 BIOINFORMATIC TOOLS TO STUDY PROTEIN S‐ACYLATION

### 4.1 SwissPalm

SwissPalm serves as a centralized cross‐species database, compiling reported S‐acylation substrates from curated proteome datasets and experimental validations in the literature.^29^ SwissPalm offers a search functionality that allows users to look up any given protein and see how frequently it has been identified in acyl‐proteome datasets or validated experimentally. Swisspalm provides S‐acylation site prediction through tools like PalmPred^30^ and CSS‐Palm^31^ which facilitates comparison of protein lists with its database, supporting various filtering options. When associated to other bioinformatics tools (such as Gene Ontology), it helps in understanding how S‐acylation might influence biological pathways. However, the database has limitations, including a substantial statistical false discovery rate associated with mass spectrometry and background detection issues from hydroxylamine‐dependent protein capture. Experimental validation and determination of biological relevance have so far been limited to a small number of proteins, and proteomics studies do not directly confirm the S‐acylation of all identified hits.

### 4.2 BrainPalmSeq

BrainPalmSeq is an online database of RNA‐seq data focusing on S‐acylation regulation in the brain. It compiles gene expression information from various RNA‐seq studies, offering standardized interactive heatmaps.^32^ Users can explore expression of genes that regulate S‐acylation in the brain, including zDHHC enzymes, deacylation enzymes, and known zDHHC accessory proteins. Across various brain regions or cell types. The limitations include potential biases in the reanalyzed data, the dependency on the quality of the original RNA‐seq studies, and the complexity of accurately predicting functional outcomes based exclusively on expression patterns.

### 4.3 CellPalmSeq

CellPalmSeq is similar to BrainPalmSeq, and focuses on human single‐cell and whole RNA‐seq data from numerous cancer and non‐human cell lines.^33^ For cancer research, it helps identify S‐acylation and deacylation enzymes enriched in cancer cell lines, potentially informing targeted cancer therapies. It also offers interactive heatmaps to compare gene expression patterns related to S‐acylation regulation across datasets. Bar charts allow comparisons for gene expression across various tissues, cell types, or cancer cell lines. Limitations may include biases in the datasets analyzed, variability in RNA‐seq data quality across different studies, and challenges in directly translating gene expression into functional implications without additional experimental validation.

## 5 S‐ACYLATION AND DE‐ACYLATION ENZYMES INVOLVED IN CANCER

In this section, we review recent studies linking zDHHC and APT enzymes to cancer as candidate oncoproteins, tumor suppressors, or prognostic markers.

### 5.1 zDHHC1

zDHHC1 was identified as potential tumor suppressor frequently silenced by epigenetic modifications like promoter methylation in cancer tissues and cell lines.^33^ In MCF7 breast and HONE1 nasopharyngeal carcinoma cell lines, restoring zDHHC1 expression by methylation inhibition or by ectopic expression promoted apoptosis and cell cycle arrest, reversed epithelial to mesenchymal transition (EMT) and stemness biomarkers, and decreased tumor growth and metastasis in xenografts. zDHHC1 downregulation decreased ER stress and pyroptosis markers GRP78, CHOP, NLRP3, and IL‐1β, indicating that zDHHC1 promotes proinflammatory cell death. Subsequently, zDHHC1 was identified as a tumor suppressor in cohorts of prostate cancer (PCa) patients.^34^ Among six pyroptosis‐related genes, zDHHC1 was negatively associated with a higher probability of biochemical recurrence, immune infiltration, and degraded clinicopathological features. zDHHC1 downregulation promoted the proliferation and migration of DU‐145 and PC‐3 cells and their growth and metastasis in xenografts, while its overexpression promoted pyroptosis, confirming the implication of this isoform in proinflammatory‐driven cell death.

### 5.2 zDHHC2

Loss of zDHHC2 heterozygosity was associated with early metastatic recurrence after liver transplantation in a cohort of 40 patients with hepatocellular carcinoma (HCC).^35^ zDHHC2 expression was decreased in HCC samples and cell lines while zDHHC2 overexpression inhibited proliferation, migration, and invasion in HCC cell lines, suggesting that zDHHC2 acts as tumor suppressor.^35^ Accordingly, zDHHC2 expression was reduced in gastric tumor tissues from patients with gastric adenocarcinoma and was associated with lymph node metastasis and unfavorable prognosis.^36^ In contrast, zDHHC2 was upregulated in renal cancer tissues and cell lines resistant to tyrosine kinase inhibitors (TKIs).^37^ TKIs suppress the vascular endothelial growth factor (VEGF) signaling pathway, angiogenesis, and the progression of malignant tumors. Sunitinib, a TKI, has been approved as a first‐line targeted agent for clear cell renal cell carcinoma (ccRCC),^38^ and resistance to sunitinib has been documented in this context.^39^ Amongst the pathways involved in resistance to TKIs is lipid metabolism.^40^ In cellular and mouse models of ccRCC, zDHHC2‐mediated S‐acylation contributed to sunitinib resistance by promoting the PM localization of acylglycerol kinase (AGK) and the subsequent activation of the AKT‐mTOR pathway.^37^

### 5.3 zDHHC3

The integrin α6β4, implicated in tumor progression,^41^ metastasis,^42^ and angiogenesis,^43^ was reported to be S‐acylated by the enzyme zDHHC3 in breast and PCa cell lines.^44^ zDHHC3 downregulation decreased integrin signaling without affecting other integrins and reduced β4 phosphorylation and cell surface expression by promoting its endosomal degradation. zDHHC3 was linked to the programmed death‐ligand 1 (PD‐L1) axis in colon cancer cell lines.^45^ PD‐L1 blockade has revolutionized anticancer immunotherapy^46^ and post‐translational modifications control PD‐L1 turnover, affecting the clinical response to anti‐PD‐1/PD‐L1 therapies. zDHHC3 reportedly S‐acylates PD‐L1 at Cys272, preventing its lysosomal degradation. Inhibition of PD‐L1 S‐acylation by 2‐bromo‐palmitate or zDHHC3 silencing activated antitumor immunity in vitro and in mice models. A competitive inhibitor of PD‐L1 S‐acylation reduced PD‐L1 expression in tumor cells, enhancing T‐cell immunity. Reduced zDHHC3 expression correlated with unfavorable outcomes and high expression with better prognosis in kidney clear cell carcinoma (KIRC),^47^ a disease with poor prognosis and limited therapeutic options.^48^ Genes linked to high zDHHC3 expression in KIRC were mainly involved in ion transport and included the sodium‐hydrogen exchanger SLC9A2. In Caki‐2 cells zDHHC3 ablation decreased SLC9A2 S‐acylation and prevented apoptosis. zDHHC3 is linked to reduced patient survival in breast cancer (BrCa) and upregulated in metastatic tumors.^49^ zDHHC3 downregulation decreased the size of primary tumor and metastasis in MDA‐MB‐231 xenografts and promoted oxidative stress, focal adhesion kinase (FAK) and STAT3 activation^50^ and the secretion of senescence‐associated proteins.^51^ Additionally, zDHHC3‐suppressed tumors exhibited increased recruitment of innate immune cells associated with senescent tumor clearance.^52^

### 5.4 zDHHC4

zDHHC4 upregulation correlates with tumor grade and poor prognosis in glioblastoma (GBM)^53^ and was linked to glycogen synthase kinase 3β (GSK3β), an enzyme involved in malignant progression.^54^, ^55^ zDHHC4‐mediated S‐acylation of GSK3β at Cys14 enhanced resistance to temozolomide, enriching GBM stem cell populations and increasing their self‐renewal capacities by promoting STAT3 interactions with the histone methyltransferase EZH.^56^

### 5.5 zDHHC5

zDHHC5 expression was also linked to stemness and malignant growth in glioma. In a cohort of sixty patients bearing p53 mutations associated with resistance to therapy, zDHHC5 expression correlated with p53 mutations.^57^ Mutated p53 transcriptionally upregulated zDHHC5 along with the nuclear transcription factor NF‐Y, enhancing the self‐renewal capacity and tumorigenicity of glioma stem cells (GSCs). Downregulation of zDHHC5 decreased neurosphere formation and invasiveness. EZH2 was identified as a substrate for zDHHC5, with cysteines 571 and 576 being required for EZH2 S‐acylation. Another study linked zDHHC5 to glioma via the S‐acylation of FAK,^58^ an enzyme that promotes cell migration and invasion.^59^ zDHHC5 knockdown disrupted FAK S‐acylation and membrane distribution, impairing proliferation and invasion of glioma cancer cells, while a catalytically inactive zDHHC5 Cys134S mutant reduced glioma xenografts growth. Elevated zDHHC5 protein expression also correlated with poor survival in non‐small cell lung cancer (NSCLC).^60^ Downregulation of zDHHC5 inhibited the growth of NSCLC lines without affecting normal human bronchial epithelial lines, reducing cell proliferation, colony formation, and invasion. These effects were reversed by re‐expression of wild‐type zDHHC5 but not of the catalytically inactive mutant and tumor xenograft formation was also decreased upon zDHHC5 downregulation.

### 5.6 zDHHC6

The ER‐resident zDHHC6 was reported to S‐acylate the oncoprotein NRAS. Artemisinin (ART), an antimalarial compound with potent anticancer properties, was shown to covalently bind and inhibit zDHHC6, suppressing NRAS S‐acylation and reducing the proliferation of HeLa and MCF7 cells.^61^

### 5.7 zDHHC7

zDHHC7 expression is reduced in human PCa tissues and this decline correlates with negative clinical outcomes.^62^ In the androgen receptor positive PCa cell lines LNCaP and 22Rv1, zDHHC7 overexpression inhibited the transcription of genes involved in the cell cycle and steroid biosynthesis pathways. zDHHC7 depletion increased the oncogenic properties of PCa cells, an effect reversed by zDHHC7 re‐expression. In contrast, zDHHC7 expression is associated with poor prognosis in liver cancer^63^ in a pathological loop involving the transcription factor STAT3. In HepG2 cells, zDHHC7 S‐acylates STAT3 at Cys108, increasing the expression of STAT3 target genes, including HIF1α that in turn promotes zDHHC7 expression in a positive feedback loop.^64^ Pharmacological inhibition of zDHHC7 reduced STAT3 S‐acylation, decreased HIF1α levels, and inhibited HCC cell proliferation in vivo.

### 5.8 zDHHC8

zDHHC8 was implicated in mesothelioma, a cancer with poor prognosis due to its intrinsic radioresistance.^65^ zDHHC8 knockdown improved the benefits of X‐irradiation in mesothelioma cell lines,^66^ increasing apoptotic and micronucleic cells arrested in the G2/M checkpoint, and suppressed tumor growth, reduced cell proliferation, and promoted apoptosis in tumor‐bearing mice exposed to X‐irradiation. zDHHC8 knockdown thus enhances the efficacy of radiation therapy in malignant mesothelioma by inducing cell cycle arrest. In GBM, zDHHC8 was reported to regulate the cystine/glutamate antiporter SLC7A11 implicated in ferroptosis, an iron‐dependent type of programmed cell death characterized by the accumulation of lipid peroxides.^67^ S‐acylation of SLC7A11 at Cys327 by zDHHC8 was shown to prevent SLC7A11 polyubiquitination and degradation,^68^ and phosphorylation of zDHHC8 at Ser299 by AMPKα1 to enhance zDHHC8‐SLC7A11 interactions, further promoting SLC7A11 S‐acylation and stabilization. zDHHC8 knockdown increased ferroptosis in GBM cells, leading to impaired cell survival, rescued by ectopic SLC7A11 expression. In GBM patients, zDHHC8 expression correlated with SLC7A11/AMPKα1 expression in glioma samples and high co‐expression levels were associated with poor prognosis.

### 5.9 zDHHC9

zDHHC9 expression is upregulated in colon cancer and correlates with bad prognosis.^69^ In human DLD‐1 and mouse MC38 colon cancer cell lines, zDHHC9 expression promoted IFN‐γ‐induced JAK/STAT1 activation and PD‐L1 upregulation. zDHHC9 knockdown promoted colon cancer cell proliferation in vitro but decreased tumor growth in vivo, increasing immune cell infiltration and enhancing T cell‐mediated cytotoxicity. zDHHC9 silencing with nanoparticles also induced a considerable regression of pancreatic tumors and extended the survival of mice with transplantable pancreatic tumors,^70^ enhancing inflammation and infiltration of anti‐tumor immune effector cells and boosting anti‐PD‐L1 immunotherapy.

### 5.10 zDHHC11

The zDHHC11 gene is atypical as it generates three distinct transcripts: a standard mRNA (pczDHHC11), a linear long non‐coding RNA (lnczDHHC11), and a circular RNA (circzDHHC11), all sharing 18 miR‐150 binding sites implicated in tumor suppression. The three zDHHC11 transcripts were proposed to act as microRNA sponge to promote the growth of Burkitt lymphoma (BL),^71^ a highly aggressive B‐cell lymphoma, by releasing the transcription factor c‐MYB from miR‐150‐mediated repression.^72^, ^73^ Knockdown of circzDHHC11 inhibited the growth of BL cell lines but ectopic expression of circzDHHC11 had no effect on cell growth and did not rescue the growth inhibition enforced by miR‐150 overexpression. Moreover, knockdown of circzDHHC11 still inhibited growth of BL cells lacking the miR‐150 binding site region, indicating that circzDHHC11 promotes the growth of BL independently of miR‐150 sequestration, likely at the post‐transcriptional level.

### 5.11 zDHHC12

zDHHC12 is upregulated in high‐grade serous ovarian cancer (HGSOC) and zDHHC12 knockdown or pharmacological inhibition with 2‐bromo‐palmitate sensitized HGSOC cells to cisplatin treatment in ovarian xenografts and in ascites‐derived organoid of platinum‐resistant ovarian cancer.^74^ zDHHC12 was shown to mediate the S‐acylation of membrane claudin‐3 (CLDN3), a tight junction protein that positively correlates with ovarian cancer progression.^75^ S‐acylation of CLDN3 by zDHHC12 on three juxta‐membrane cysteine residues (Cys181, Cys182, and Cys184) promotes CLDN3 PM localization and protein stability. In an ovarian cancer cell line, CLDN3 or zDHHC12 silencing disrupted CLDN3 S‐acylation and abolished tumorigenesis.^76^

### 5.12 zDHHC13

A protective role for zDHHC13 in skin cancer was uncovered by a spontaneous mutation in the zDHHC13 gene (Zdhhc13luc), leading to a premature stop codon and the production of a truncated zDHHC13 protein with loss‐of‐function.^77^ Homozygous mutant mice^Zdhhc13luc/Zdhhc13luc^ developed hypotrichosis and skin abnormalities, including hyperplasia, hyperkeratosis, and increased epidermal thickness. The animals also exhibited higher susceptibility to skin carcinogenesis compared to wild‐type littermates, as evidenced by highly proliferative keratinocytes and accelerated transition through epidermal layers following exposure to tumor‐promoting agents and acute UVB exposure. The skin phenotype correlated with constitutive NF‐κB activation and enhanced neutrophil elastase secretion, factors associated with carcinogenesis.^78^ zDHHC13 was further shown to S‐Acylate the melanocortin‐1 receptor (MC1R),^79^ a melanoma gene predictor associated with red hair color and DNA damage repair.^80^, ^81^ zDHHC13 phosphorylation by AMPK at Ser208 enhanced MC1R S‐acylation, inhibiting UVB‐induced transformation of human melanocytes in vitro and delaying melanoma development in mice.

### 5.13 zDHHC14

zDHHC14 was identified as a tumor suppressor in PCa.^82^ A common small deletion of zDHHC14 was found in testicular germ cell tumors, predominantly in heterozygous form. zDHHC14 RNA and protein levels were reduced in primary tumors and in a panel of PCa cell lines (PC‐3, DU‐145, 22RV1, LNCaP, and VCaP). Overexpression of zDHHC14 decreased viability of 22RV1 cells and suppressed tumor growth in a xenograft model through induction of apoptosis.

### 5.14 zDHHC15

High zDHHC15 expression was reported in glioma‐associated datasets, correlating with malignant phenotypes and poorer prognosis,^83^ an association confirmed by immunohistochemistry of glioma samples from human patients. zDHHC15 knockdown suppressed the proliferation and migration of U87 and U251 glioma cells, reducing cyclins B1/D and MMP2/9 expression, while zDHHC15 overexpression had opposite effects that were linked to STAT3 signaling.

### 5.15 zDHHC16

High zDHHC16 expression was reported in NSCLC tissues and cell lines and associated with epigenetic m6A methylation, as silencing of methyltransferases Si‐METTL3 or si‐METTL14 decreased zDHHC16 gene expression.^84^ zDHHC16 upregulation increased glucose consumption and lactate excretion in NSCLC cells, a Warburg effect that promoted cancer cell growth and migration, and reduced CREB ubiquitination, preventing ferroptosis. Conversely, zDHHC16 silencing enhanced CREB ubiquitination, identifying CREB as a zDHHC16 target in NSCLC.

### 5.16 zDHHC17

zDHHC17 expression increases progressively along with MAP2K4 expression in glioma samples progressing from grades I to IV and correlates with poor survival.^85^ zDHHC17 knockdown inhibited cell proliferation by promoting cell cycle arrest while zDHHC17 overexpression rescued cell cycle progression. zDHHC17 was further reported to interact with MAP2K4 and p38/JNK, enhancing malignant progression, the zDHHC17‐MAP2K4‐JNK/p38 signaling axis contributing to GSC enrichment and increasing their self‐renewal capacity.

### 5.17 zDHHC18

zDHHC18 was shown to S‐Acylate malate dehydrogenase 2 (MDH2), an enzyme of the TCA cycle, whose S‐acylation levels were enhanced in samples from patients with high‐grade serous ovarian cancer.^86^ Silencing zDHHC18 suppressed MDH2 S‐acylation, reducing mitochondrial respiration and proliferation of ovarian cancer cell lines. Re‐expression of wild‐type MDH2, but not of its S‐acylation‐deficient Cys138S mutant, restored mitochondrial respiration and enhanced the growth and clonogenicity of ovarian cancer cells in vitro and in nude mice injected with MDH2‐knockout A2780 cells.

### 5.18 zDHHC19

zDHHC19 upregulation correlates with poor prognosis in osteosarcoma (OS) datasets and OS cell lines.^87^ zDHHC19 silencing inhibited proliferation, invasion, and migration of OS cells and suppressed OS growth and lung metastasis in xenografts. zDHHC19 is a target of the tumor suppressor miR‐940 and zDHHC19 overexpression mitigated the suppression of proliferation, migration, and invasion induced by miR‐940. The pro‐oncogenic effects of zDHHC19 involved the Wnt/β‐catenin pathway, suggesting that a miR‐940/zDHHC19 axis regulates the Wnt/β‐catenin pathway in OS.

### 5.19 zDHHC20

zDHHC20 was linked to metastatic growth of pancreatic ductal adenocarcinoma (PDAC).^88^ In a short hairpin RNA screen, *zdhhc20* silencing impaired the metastatic potential but not the proliferation of PDA530Met and FC1199 PDAC mouse cell lines while in a genetically engineered PDAC mouse model, z*dhhc20* disruption delayed liver and lung metastases without impacting primary tumor growth. The tumorigenicity of z*dhhc20*‐deficient cells increased in immunocompromised mice and in mice depleted of NK cells, linking their oncogenic potential to defective innate immunity, and cell surface proteins potentially engaging in interactions with NK cells were identified by zDHHC20 substrate profiling.

### 5.20 zDHHC21

zDHHC21 was identified in a post‐translational protein modifications screen as a regulator of oxidative phosphorylation (OXPHOS) in acute myeloid leukemia (AML), and its substrate identified as the mitochondrial enzyme adenylate kinase 2 (AK2).^89^ Chemical inhibition or genetic suppression of zDHHC21 weakened the stemness potential and induced the myeloid differentiation of drug‐resistant leukemia stem cells, which rely on OXPHOS for survival. zDHHC21 depletion or inhibition prevented AK2 localization to the mitochondria and decreased OXPHOS and ATP production, inhibiting the growth and viability of AML cells and stem cells without impacting normal hematopoietic cells. zDHHC21 inhibition reduced the growth of patient‐derived AML blasts injected in mice and sensitized AML blasts to the cytotoxic effects of chemotherapy in xenograft model of relapsed/refractory leukemia, highlighting the potential of zDHHC21 as a potential therapeutic target in AML.

### 5.21 zDHHC22

zDHHC22 expression was found to be reduced in oestrogen receptor (ER) negative BrCa specimens and cell lines, an effect attributed to the epigenetic methylation of its promoter, and low zDHHC22 expression correlated with good prognosis in BrCa patients.^90^ Ectopic expression of WT, but not of S‐acylation‐deficient zDHHC22, reduced the proliferation of ER‐negative BrCa cell lines, enhanced mTOR S‐acylation, decreased AKT signaling, and restored MCF‐7R cells sensitivity to oestrogen therapy.

### 5.22 LYPLA1

The de‐acylation enzyme LYPLA1 (aka APT1) was reported to be highly expressed in the NSCLC cell line SPC‐A‐1 and in other lung cancer cell lines.^91^ LYPLA1 silencing inhibited the proliferation, migration, and invasion of the NSCLC cells and was associated with increased E‐cadherin expression and decreased expression of the mesenchymal markers N‐cadherin, vimentin, and SNAIL.

### 5.23 PPT1

The PPT1 was identified by photoaffinity pulldown as a target of a chloroquine derivative preventing lysosomes acidification and reducing melanoma cells viability and tumor growth in vivo,^92^ and was subsequently involved in HCC resistance to the kinase inhibitor sorafenib.^93^ High PPT1 levels in HCC tissues correlated with poor prognosis and PPT1 was upregulated in sorafenib‐resistant cell lines. In xenografts, PPT1 expression was associated with immune infiltration and the chloroquine derivative, presumably acting via PPT1, enhanced the anti‐tumor immune response by promoting dendritic cell maturation and T cell activation. Ezurpimtrostat (aka GNS561), a small molecule accumulating in lysosomes, was also reported to inhibit PPT1 and its potential benefits for patients with advanced liver cancer are currently under evaluation.^94^

### 5.24 PPT2

PPT2 expression was found to be reduced in ccRCC samples and low levels correlate with poor survival,^95^ whereas in ccRCC cell lines PPT2 overexpression reduced EMT, cell migration, and invasion.

### 5.25 ABHD17

ABHD17 was found by dual pulse‐chase metabolic labeling (17‐ODYA) to be required for the de‐palmitoylation of the oncoprotein N‐Ras, while APT1 and APT2 were dispensable.^20^ Overexpression of ABHD17A but not of catalytically dead (Ser211A) or N‐truncated cytosolic mutants redistributed N‐Ras away from the PM, as did mutation of N‐Ras palmitoylated residue Cys181 or pharmacological inhibition of S‐acylation. Knockdown of ABHD17 isoforms inhibited N‐Ras palmitate turnover These data indicate that ABHD17 removes palmitate from N‐Ras, altering its subcellular localization. ABHD17C was identified in the PDAC database as a potential biomarker for predicting prognosis and response to anti‐PD1 therapy in pancreatic cancer patients.^96^ ABHD17C overexpression increased glycolysis in pancreatic cancer cell lines and promoted their growth and resistance to anti‐PD1 therapy when infected in mice, a phenotype associated with increased infiltration of myeloid‐derived suppressor cells and reduced cytotoxic T cells infiltration, suggesting that ABHD17C promotes the formation of an acidic, immunosuppressive environment.

## 6 CA^2+^ TRANSPORT PROTEINS IN CANCER AND THEIR REGULATION BY S‐ACYLATION

Since the discovery by Sydney Ringer of the role of calcium ions in cardiac contraction in 1883,^97^ numerous studies have highlighted the crucial role of Ca^2+^ as intracellular second messengers. In response to homeostatic or environmental cues, the free intracellular Ca^2+^ concentration, maintained at nanomolar levels by pumps and exchangers, increases to micromolar levels due to the opening of Ca^2+^ channels in the PM and in the membrane of intracellular Ca^2+^ storing organelles.^98^ The cytosolic Ca^2+^ elevations are encoded in time, space, amplitude, and frequency, and are decoded by specific effector proteins to enable the precise spatiotemporal control of cellular outcomes.^99^

**Figure 3:**
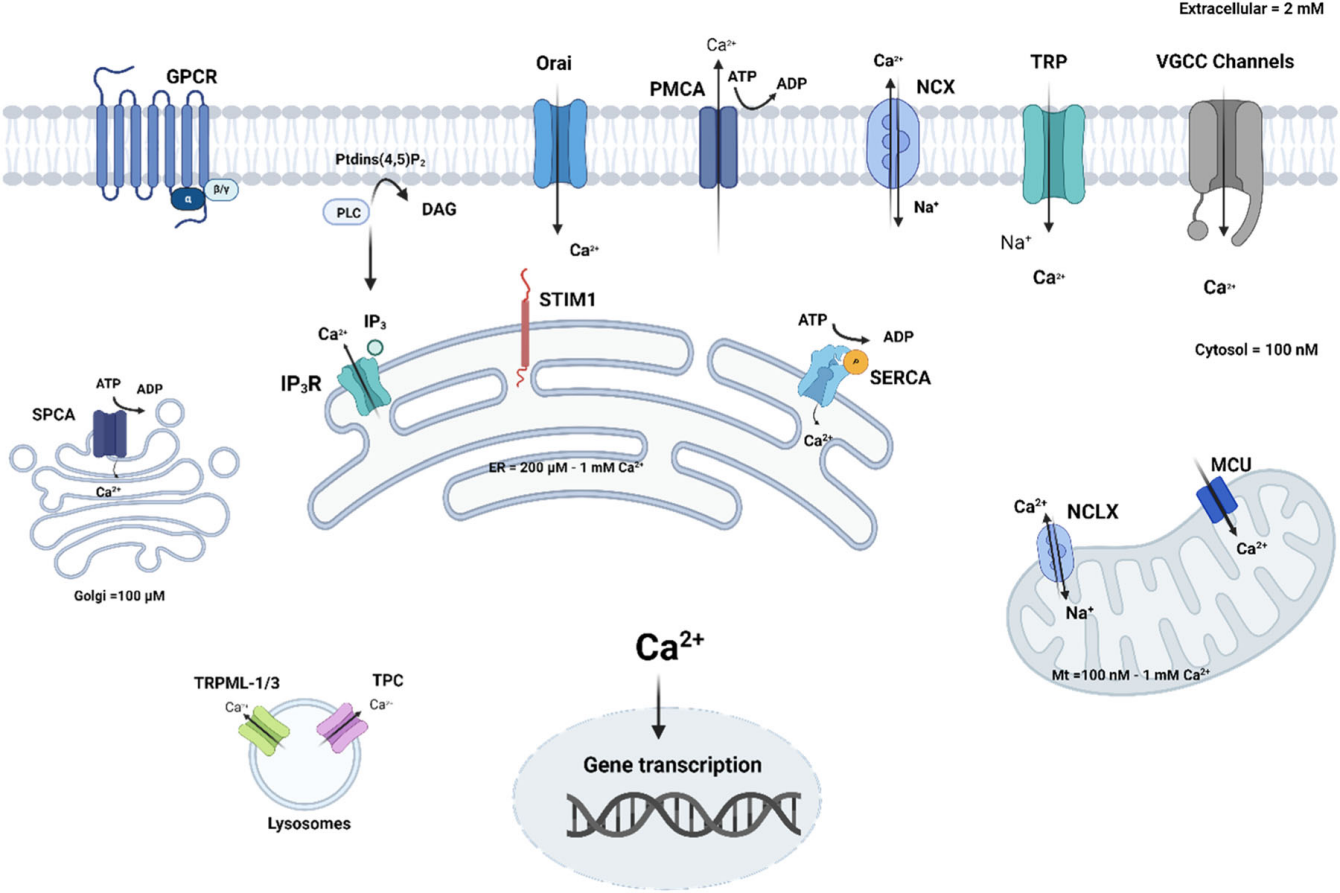
Cellular Ca^2+^ homeostasis. Ca^2+^ second messengers in the cell cytosol originate either from the extracellular milieu or from internal stores. Ca^2+^ ions cross the PM barrier across three major classes of Ca^2+^‐permeable channels: VGCC, TRP and Orai channels, and can be released by the opening of intracellular channels such as IP3R on the ER. Ca^2+^‐ATPases pump Ca^2+^ ions into organelles or into the extracellular space to maintain a low cytosolic Ca^2+^ concentration. Three Ca^2+^‐ATPases have been identified, located in ER/SR (SERCA pump), Golgi network (SPCA pump), and in the PM (PMCA pump). Ca^2+^ can be extruded at the PM via the sodium/calcium exchanger NCX1 and taken up by mitochondria via the MCU. DAG, diacylglycerol; ER, endoplasmic reticulum; GPCR, G protein‐coupled receptor; IP3R, inositol 1,4,5‐trisphosphate receptor; MCU, mitochondrial Ca^2+^ uniporter; Mt, mitochondria; NCLX, Na^+^/Ca^2+^/Li^+^ exchanger; NCX, Na^+^/Ca^2+^ exchanger; PLC, phospholipase C; PMCA, plasma membrane Ca^2+^ ATPase; PtdIns(4,5)P2, phosphatidylinositol 4,5‐bisphosphate; RTK, receptor tyrosine kinase; SERCA, sarco‐/endoplasmic reticulum Ca^2+^ ATPase; SPCA, secretary pathway Ca^2+^ ATPase; STIM, stromal interaction molecule; TPC, two‐pore channel; TRPML, transient receptor potential Mucolipin. The figure was created with www.biorender.com.

In the PM, three major classes of Ca^2+^‐permeable channels have been identified, each with distinct characteristics (Figure 3). Voltage‐gated Ca^2+^ channels (VGCCs) include CaV1 (L‐type Ca^2+^ currents: α1S, α1C, α1D, and α1F), CaV2 (P/Q‐type, N‐type, and R‐type Ca^2+^ currents: α1A, α1B, and α1E), and CaV3 (T‐type Ca^2+^ currents: α1G, α1H, and α1I).^100^ Transient receptor potential (TRP) channels comprise canonical (TRPC1‐7), vanilloid (TRPV1‐6), melastatin (TRPM1‐8), ankyrin; TRPMA1‐3), polycystin (TRPP1‐3 and 5), and mucolipin (TRPML1‐3) subfamilies, displaying a greater diversity in activation mechanisms and selectivity than any other group of ion channels.^101^ Ca^2+^ Release‐Activated Ca^2+^ (CRAC) channels work through a mechanism known as store‐operated Ca^2+^ entry (SOCE). Upon Ca^2+^ store depletion, ER‐resident stromal interaction molecule (STIM [STIM1 and STIM2)) proteins are redistributed and interact with Orai (Orai1, Orai2 and Orai3) proteins on the PM, enabling Ca^2+^ influx.^102^

In organelles, the opening of intracellular Ca^2+^ release channels such as inositol 1,4,5‐trisphosphate (IP3) receptors (IP3Rs) or ryanodine receptors (RYR) on the ER increase cytosolic Ca^2+^ levels (Figure 3). IP3 is generated by the engagement of G protein‐coupled receptors (GPCRs) or receptor tyrosine kinases, such as the epidermal growth factor receptor (EGFR), coupled to the phospholipase Cβ (PLCβ) and PLCγ isoforms.^103^ In lysosomes and other acidic organelles, two‐pore channels (TPCs) release Ca^2+^ in response to NAADP (nicotinic acid adenine dinucleotide phosphate). Restoration of basal Ca^2+^ levels is performed by Ca^2+^‐ATPases, which pump cytosolic Ca^2+^ out of cells or back into storage organelles. Three Ca^2+^‐ATPases have been identified, located in the membranes of the ER/SR (sarco‐/endoplasmic reticulum Ca^2+^‐ATPase [SERCA] pump), of the Golgi network (Secretary pathway Ca^2+^ ATPase [SPCA] pump), and in the PM (plasma membrane Ca^2+^ ATPase [PMCA] pump). In addition to the Ca^2+^‐ATPases, Ca^2+^ extrusion through the PM can occur via the sodium/calcium exchanger NCX1.^104^ Ca^2+^ buffering is also achieved by the mitochondrial Ca^2+^ uniporter (MCU) complex, which transports Ca^2+^ into mitochondria when cytoplasmic Ca^2+^ is rapidly elevated.^105^

**Figure 4:**
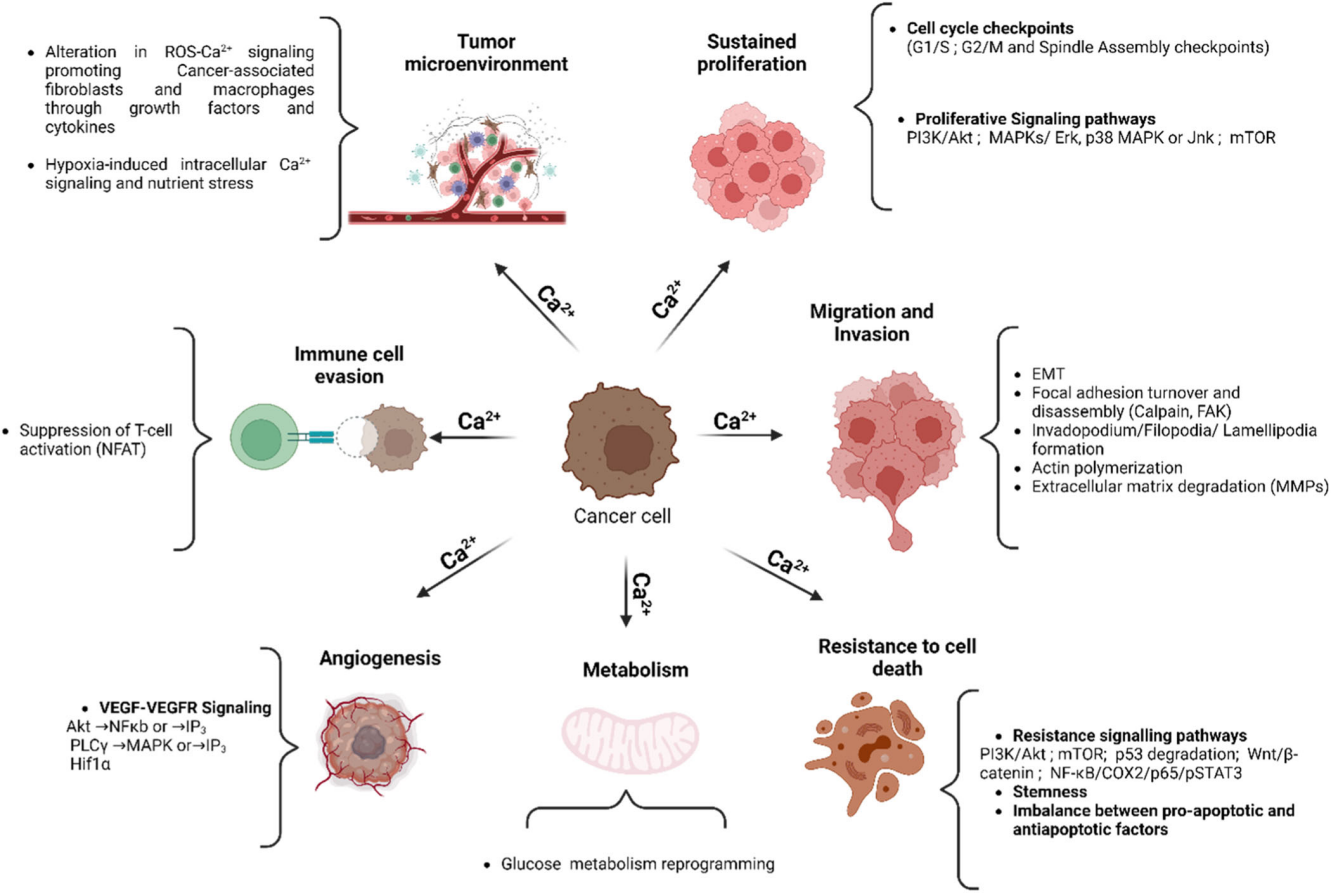
Ca^2+^ signaling in cancer. Ca^2+^ controls proliferation by regulating cell cycle machinery components (cyclins, Cyclin dependent Kinases [CDK], CDK inhibitors, centrosome cycle). This regulation occurs either directly through Ca^2+^‐binding proteins or indirectly via transcription factors (NFAT, or immediate early genes such as JUN, Myc, and FOS), and Ca^2+^‐dependent oncogenic pathways such as Ras/ERK and PI3K/Akt.^109^, ^110^ Ca^2+^ ions regulate survival pathways,^111^ stemness,^112^ and death factors (e.g., BAX/BCL family proteins).^113^ Ca^2+^ signaling also influences migration and invasion through epithelial‐mesenchymal transition by modulating specific gene expression markers and controlling interactions with the extracellular matrix. At the leading edge, Ca^2+^ triggers lamellipodia formation, focal adhesion assembly, and contact with the matrix, while at the uropod it facilitates focal adhesion disassembly through Ca^2+^‐sensitive protease calpain.^114^, ^115^ Ca^2+^ regulates tumor cell metabolism, particularly glucose metabolism and metabolic energetics.^116^, ^117^ It is also involved in angiogenesis by promoting vascularization of the primary tumor and recruitment of endothelial cells to the tumor site.^118^ Moreover, Ca^2+^ overload in T‐cells impairs their activation and cytotoxicity (through NFAT), enabling evasion of immunosurveillance.^119^ Alterations in ROS and Ca^2+^ levels in the tumor microenvironment generated by hypoxia and nutrient stress can recruit tumor‐associated fibroblasts and macrophages^120^, ^121^ The figure was created using www.biorender.com. ROS, reactive oxygen species.

**Figure 5:**
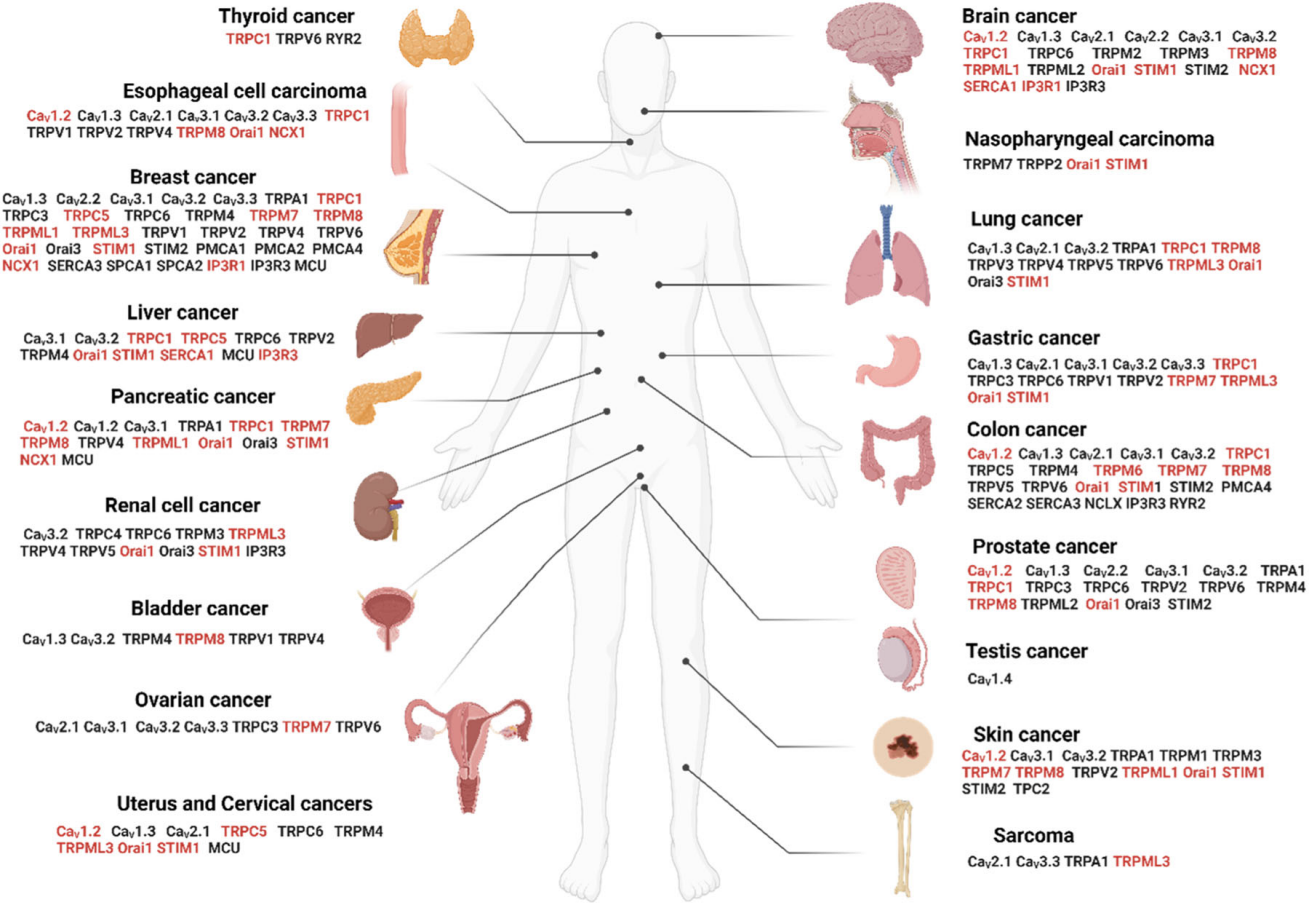
Ca^2+^ transport proteins involved in cancer (solid tumors). The implication of Ca^2+^ transport proteins in cancer has been previously documented in several studies recently reviewed. Readers are referred to: VGCC^122^, ^123^, ^124^, ^125^; TRP channels^122^, ^125^, ^126^, ^127^; Orai‐STIM channels^128^, ^129^, ^130^, ^131^, ^132^; PMCA^125^, ^133^, ^134^; NCX1^135^; SERCA^125^, ^136^, ^137^; SPCA^138^; MCU^139^; NCLX^139^, ^140^, ^141^; IP3R^142^, ^143^, ^144^; RYR.^145^, ^146^ Transporters highlighted in red have been biochemically documented to be S‐acylated. IP3R, inositol 1,4,5‐trisphosphate receptor; MCU, mitochondrial Ca^2+^ uniporter; NCLX, Na^+^/Ca^2+^/Li^+^ exchanger; NCX, Na^+^/Ca^2+^ exchanger; PMCA, plasma membrane Ca^2+^ ATPase; RYR, ryanodine receptor; SERCA, sarco‐/endoplasmic reticulum Ca^2+^‐ATPase; SPCA, secretary pathway Ca^2+^ ATPase; STIM, stromal interaction molecule; TRP, transient receptor potential; VGCC, voltage‐gated Ca^2+^ channels. The figure was created using www.biorender.com.

Ca^2+^ signals regulate cellular pathways involved in cancer onset or progression (Figure 4), including gene expression, cell proliferation, migration, and resistance to death signals.^106^ In the tumor microenvironment, alterations in Ca^2+^ signaling are associated with malignant transformation, immune cell evasion^107^ and resistance to cancer therapy.^108^ Several classes of Ca^2+^ transporters, predominantly ion channels, are associated with the development and progression of human cancers and their implication is graphically illustrated along with relevant references in Figure 5. Table S2 summarizes the current knowledge on the S‐acylation of Ca^2+^ transport proteins established by biochemical assays and predicted by the SwissPalm algorithm (criteria CSS‐Palm 4.0: High confidence and PalmPred: High confidence score >0.4). Cross‐referencing these datasets identifies NCX1, TRPC1, TRPC5, TRPM7, TRPM8, TRPML3, ORAI1/STIM1 and IP3R1 as cancer‐associated Ca^2+^ transporters regulated by S‐acylation via known enzyme(s). Studies exploring the potential role of the S‐acylation of these Ca^2+^ transporters in cancer are discussed in the next chapter.

## 7 DOES S‐ACYLATION OF CA^2+^ TRANSPORTERS CONTRIBUTE TO CANCER?

According to the studies summarized above, all the enzymes mediating the addition and removal of lipids by S‐acylation are implicated in cancer (Table S1) and several PM and intracellular Ca^2+^ channels, pumps, and exchangers are associated with solid tumors (Figure 5). This is not unexpected given the multiplicity of cellular effector functions impacted by S‐acylation and by Ca^2+^‐dependent biochemical reactions. An increased expression of the genes coding for acyltransferases and thioesterases is a marker of bad prognostic, indicating that the turnover of these enzymes, rather than the lipidation state of their target proteins, limits cancer progression. On the other hand, an increased expression of the genes coding for Ca^2+^ transport proteins are usually associated with unfavorable outcomes, indicating that augmenting Ca^2+^ fluxes across cellular membranes promotes cancer progression. Notably, an increased S‐acylation augments Ca^2+^ fluxes mediated by all the transporters that have been biochemically verified to be S‐Acylated, except NCX1, whose function is decreased by S‐acylation. Since NCX1 usually extrudes Ca^2+^ ions to restore basal cytosolic Ca^2+^ levels, the picture that emerges from the biochemical and functional studies is that the S‐acylation of cellular Ca^2+^ transporters elevate the cytosolic Ca^2+^ concentration to levels that promote cancer progression. Of note, NCX1 was reported to operate in reverse mode in solid and hypoxic tumors,^147^, ^148^, ^149^ and it will be interesting to establish the S‐acylation status of NCX mediating Ca^2+^ uptake in this context.

S‐acylation has two major impacts on the function of Ca^2+^ transport proteins. First, the enhanced affinity of acylated proteins for ordered lipids promotes their accumulation in membrane domains rich in cholesterol and sphingolipids, thereby altering the distribution of transporters in membranes and their interactions with regulatory proteins or lipids. Second, the addition of acyl chains can directly alter the conformation of TM domains, thereby affecting the gating, transport rates, and regulation of Ca^2+^ transporters. Both effects contribute to Ca^2+^ alterations in cancer models. Accumulation of SK3/Orai1 or TRPC1/SK3/Orai1 complexes in lipid rafts promotes Ca^2+^ entry and the migration or breast or colon cancer cells, while disruption of lipid rafts impairs Ca^2+^ entry and cell migration.^150^, ^151^ S‐acylation promotes the accumulation of Orai1 in cholesterol‐rich lipid domains^152^ and might contribute to malignancy by modulating its Ca^2+^ channel activity.^153^ S‐acylation also controls the affinity for lipids of the SOCE regulatory protein STIM1, which interacts with PM phospholipids via *its* exposed polybasic^154^ and SOAR domains.^155^ STIM1 is S‐Acylated at Cys437 within SOAR, and this lipidation promotes STIM1 PM translocation and interactions with Orai1.^156^ The coordinated S‐acylation of Orai1 and STIM1 by zDHHC and APT enzymes might recruit both proteins to the same lipid domain to optimize Ca^2+^ fluxes, as suggested by studies linking enzymes involved in STIM‐ORAI1 S‐acylation/de‐acylation and cancer (Table S1).

S‐acylation enhances the activity and prevents the degradation of TRPM7^157^ and TRPC5 channels.^158^ These Ca^2+^ and Mg^2+^‐permeable channels (for TRPM7) contribute to cancer chemoresistance^112^ and promote cell proliferation, invasion and metastasis via their ion channel activity on the cell surface of cancer cells.^126^, ^159^ S‐acylation also enhance the activity of IP3R1,^160^ promoting cytosolic Ca^2+^ elevations via the release of Ca^2+^ from intracellular stores and the subsequent activation of SOCE. The Ca^2+^ released by IP3R1 is efficiently captured by mitochondria at membrane contact sites,^161^ and the dynamic S‐acylation/de‐acylation of IP3R1 promotes receptor tethering and signaling at these locations.^162^ IP3R1 S‐acylation, therefore, enhances both the cytosolic and mitochondrial Ca^2+^ concentrations and might represent a particularly risky combination. Moreover, some zDHHC enzymes are positively regulated by Ca^2+^ elevations. zDHHC21 was recently shown to be a Ca^2+^/calmodulin‐dependent enzyme critical for activation of naïve CD4^+^ T cells,^163^ in a positive Ca^2+^ feedback loop mimicking constitutive channel activation. Finally, several pathways identified in Table S1 as mediating the oncogenic effects of zDHHC enzymes are directly or indirectly influenced by intracellular Ca^2+^ levels, including PI3K/Akt,^164^ mTOR,^165^, ^166^ NF‐κB,^167^ and STAT3.^168^, ^169^ Further research should aim to clarify the mechanisms linking these enzymes and their targets to cancer progression.

## 8 CONCLUSION AND FUTURE PERSPECTIVES

Recent studies have linked cancer progression to alterations in S‐acylation enzymes, which dynamically add lipids to proteins, and changes in proteins that regulate Ca^2+^ fluxes across cellular membranes. This review examines the link between cancer progression and these alterations in S‐acylation enzymes and Ca^2+^ transporters, with a focus on the dynamic regulation of S‐acylation of Ca^2+^ channels, pumps, and exchangers. These findings suggest that lipidated Ca^2+^ transporters could promote cancer, indicating a need for further research into this signaling axis to identify new therapeutic targets for cancer treatment. Currently, inhibitors lack specificity for zDHHC enzymes, highlighting the need for selective compounds. Examples include tetrazole‐containing TTZ‐1 and TTZ‐2 compounds, which effectively inhibit zDHHC2‐mediated S‐acylation,^170^ and acrylamide‐based agents like cyanomyracrylamide, which blocks zDHHC20 activity.^171^ Additionally, targeting palmitate‐derived palmitoyl‐CoA with FASN (Fatty Acid Synthase) inhibitors such as TVB‐3166 or TVB‐3664 shows promise in inhibiting tumor growth, especially when combined with taxane‐based treatments.^172^ Furthermore, inhibitors of de‐acylation enzymes like ML‐348 and ML‐349 enhance tumor suppressor activities and induce apoptosis through mechanisms linked to ER stress and mTOR signaling^173^ which are closely associated with Ca^2+^ signaling pathways. The development of precise inhibitors requires integrating structural biology with high‐throughput screening techniques to identify compounds that can selectively target cancer cells through S‐acylation without affecting normal cellular functions, ultimately opening new avenues for more effective and less toxic cancer therapies.

## AUTHOR CONTRIBUTIONS

**Sana Kouba**: conceptualization, literature search, manuscript writing, and final editing. **Nicolas Demaurex**: supervision, manuscript writing, critical revision of the manuscript, and approval of the final version.

## CONFLICT OF INTEREST STATEMENT

The authors declare no conflicts of interest.

## ETHICS STATEMENT

None.

## Supporting information

Supporting information.

## Data Availability

None.
