# Supplementary material for: S‐acylation of Ca2+ transport proteins in cancer
Source: Chronic Dis Transl Med. 2024 Aug 14;10(4):263–80. doi: 10.1002/cdt3.146 (PMC11483607; doi:10.1002/cdt3.146)
Supplement: Supplementary file 1 — Supporting information. [file CDT3-10-263-s001.docx]

**Table 1: S-acylation and de-acylation enzymes expression and involvement in cancer**

**↑** High mRNA level is a favourable prognostic marker

↑ High mRNA level is unfavourable prognostic marker

| **Gene** | **Localization**  **(Human Protein Atlas)** | **Prognostic summary**  **(Human Protein Atlas)** | **Cancer Type and cell model** | **Cancer phenotype** | **Reference** |  |
| --- | --- | --- | --- | --- | --- | --- |
| *zDHHC1* | ER | **↑** endometrial, renal, and pancreatic cancers | MCF7 breast and HNOE1 nasopharyngeal carcinoma cell lines.  Xenograft mouse model | zDHHC1 inhibits glucose metabolism induces oxidative/ER stress leading cell to apoptosis and cell cycle arrest, repressing metastasis, and reversing EMT transition and cell stemness. | ^1^ |  |
|  |  |  | TCGA and GEO cohorts.  PC-3 and DU-145 PCa cell lines  Xenograft mouse model | zDHHC1 promotes migration, invasion, and proliferation of PCa cells *in vitro* and attenuates pyroptosis. | ^2^ |  |
| *zDHHC2* | PM, ER, Golgi | **↑** renal cancer | HCC clinical samples and HCC Hep3B, HuH-7, Bel-7402, MHCC97L, and HCCLM3 cell lines. | Low zDHHC2 expression promotes proliferation, migration, and invasion of Bel-7402 HCC cell line | ^3^ |  |
|  |  |  | Clinical samples of gastric adenocarcinoma | Low zDHHC2 expression promotes lymph node metastasis and is unfavourable prognosis in gastric adenocarcinoma patients | ^4^ |  |
|  |  |  | Clinical specimens of CcRCC and ccRCC 786-O and A498 cell lines | zDHHC2 promotes proliferation and angiogenesis through S-acylation and translocation of AGK into the PM and activation of the PI3K–AKT–mTOR signalling pathway | ^5^ |  |
| *zDHHC3* | Golgi | **↑** renal and colorectal cancers  **↑** liver cancer | PC-3 and MDA-MB-231 cancer cell lines | zDHHC3 promotes integrin-dependent signalling through Src, and β4 phosphorylation | ^6^ |  |
|  |  |  | Human CRC HCT116, LoVo, RKO, SW1116,  SW480, as well as mouse colon carcinoma MC38 | zDHHC3 S-acylates and stabilizes PD-L1 by blocking its ubiquitination and suppresses its degradation by lysosomes. zDHHC3 downregulation enhances T-cell-mediated cell death. | ^7^ |  |
|  |  |  | Kidney clear cell carcinoma Caki-2 and RCC23 cell lines | zDHHC3 promotes S-Acylation of SLC9A2, and enhances survival | ^8^ |  |
|  |  |  | Triple negative BrCa cell line MDA-MB-231 and xenograft mouse model. | zDHHC3 in MDA-MB-231 attenuates oxidative stress and cellular senescence | ^9^ |  |
| *zDHHC4* | ER | **↑** renal cancer,  **↑** head and neck, urothelial, and cervical cancers | GBM SF126R and U118MGR cell lines (temozolomide resistant) | zDHHC4 mediates GSK3β S-acylation and promotes tumorigenicity of GBM stem cells through the EZH2–STAT3 axis | ^10^ |  |
| *zDHHC5* | PM | **↑** pancreatic cancer | Human glioma specimens,  Glioma cell lines U87, D54, A172, CCF-STTG1, U373, U251, U118MGT-98G, and SWO38 and glioma stem cell cultures | zDHHC5 promotes glioma stem-like cells and tumorigenicity through S-acylation of the tumour suppressor EZH2. | ^11^ |  |
|  |  |  | Glioma U251 and T98G cells | zDHHC5 promotes the S-acylation of FAK and stabilises its membrane localization which enhances cell proliferation, cell invasion and EMT | ^12^ |  |
|  |  |  | NSCLC cell lines H1299, H358, and H2009 | zDHHC5 promotes cell proliferation, colony formation, and cell invasion of cancer cells | ^13^ |  |
| *zDHHC6* | ER | Gene product is not prognostic | MCF7 breast cancer cell line and Hela cervical cancer cells | zDHHC6 promotes the S-acylation of the oncogenic protein NRas, and its pro-proliferative signalling cascades. | ^14^ |  |
| *zDHHC7* | Golgi | **↑** renal cancer  **↑** liver cancer | LNCaP and 22RV1 PCa cell lines  Xenograft mouse model | zDHHC7 low expression decreases the oncogenic properties of PCa cells | ^15^ |  |
|  |  |  | HepG2 HCC cells and xenograft mouse model | zDHHC7 promotes S-acylation of the transcription factor STAT3 which promotes proliferation of HCC cancer cells and tumour growth *in vivo.* | ^16^ |  |
| *zDHHC8* | Golgi | **↑** renal and cervical cancers | 211H and H2052 mesothelioma cell lines | zDHHC8 enhances radioresistance and promotes survival. | ^17^ |  |
|  |  |  | U251 or T98G human glioma cancer cell lines | zDHHC8 S-acylates SLCA11 and promotes cell survival | ^18^ |  |
| *zDHHC9* | Golgi, ER | **↑** cervical, breast, and head and neck cancers | MC38 mouse and human DLD-1 colon cancer cell lines.  Mouse Xenograft model | zDHHC9 attenuates the proliferation of colon cancer cells *in vitro* but enhances their growth *in vivo*. zDHHC9 promotes IFN-γ-induced JAK/STAT1 activation and upregulated programmed death-ligand 1 (PD-L1) expression | ^19^ |  |
|  |  |  | Transplanted pancreatic tumours in mice | zDHHC9 promotes tumour progression and resistance to anti-PD-L1 immunotherapy | ^20^ |  |
| *zDHHC11* | ER | Gene product is not prognostic | Burkitt lymphoma cell lines ST486, BL41, CA46 and DG75 | circzDHHC11 transcript promotes Burkitt lymphoma cell growth | ^21^ |  |
| *zDHHC12* | Golgi, ER | Gene product is not prognostic | High‐grade serous ovarian cancer HGSOC cell lines SNU119 and OVSAHO | zDHHC12 promotes cisplatin resistance | ^22^ |  |
|  |  |  | OVCAR8, SKOV3, HaCaT, and A2780 cells  Mouse Xenograft model | zDHHC12-mediated S-acylation promotes the cell membrane localization of CLDN3 and maintains | ^23^ |  |
| *zDHHC13* | Golgi, ER | Gene product is not prognostic | Homozygous Zdhhc13luc/Zdhhc13luc mice | zDHHC13-mediated S-acylation of MC1R-RHC attenuates susceptibility to Skin Carcinogenesis | ^24^ | |
|  |  |  | Human primary melanocytes  C57BL/6J-MC1R-RHC mice | zDHHC13-mediated S-acylation of MC1R-RHC repressed UVB-induced transformation of human melanocytes *in vitro* and delayed melanoma genesis *in vivo* | ^25^ | |
| *zDHHC14* | ER | **↑** in pancreatic cancer | Prostate cancer clinical samples  PC-3, DU-145, LNCaP, VCaP, and 22RV1 cells lines | zDHHC14 is tumour suppressor in PCa. zDHHC14 reduces cell viability and promotes apoptosis | ^26^ |  |
| *zDHHC15* | Golgi | Gene product is not prognostic | U87 and U251 glioma cell lines | zDHHC15 promotes cell proliferation and migration through STAT3 signalling pathway. | ^27^ |  |
| *zDHHC16* | ER, Nuclear membrane | Gene product is not prognostic | NSCLC A549, NCL-H129, NCL-H3122 and NCL-H466 cell lines | zDHHC16 promotes cell Proliferation and Metastasis by enhancing CREB expression and inhibition of CREB Ubiquitination | ^28^ |  |
| *zDHHC17* | Golgi | **↑** renal cancer | U118MG glioma cell lines | zDHHC17 interacts with MAP2K4 and p38/JNK to promote malignant progression | ^29^ |  |
| *zDHHC18* | Microtubules | **↑** renal, liver, and glioma cancers | OVCA433, A2780 ovarian cancer cell lines  Nude mice injected with MDH2-knockout A2780 cells re-expressing MDH2WT or MDH2C138S | zDHHC18-mediated S-acylation of Malate dehydrogenase 2 (MDH2) promotes proliferation | ^30^ |  |
| *zDHHC19* | Not available | Gene product is not prognostic | 143B and MG63 OS cancer cell lines  Xenograft model | zDHHC19 promotes proliferation, invasion and migration of OS *in vitro* and suppress tumorigenicity and lung metastasis *in vivo* through the wnt/β-catenin pathway | ^31^ |  |
| *zDHHC20* | PM | **↑** renal and pancreatic cancers | PDAC human PDA530Met and mouse FC1199 cell lines  Genetically engineered PDAC | zDHHC20 promotes migration *in vitro* and metastasis *in vivo* | ^32^ |  |
| *zDHHC21* | PM | **↑** urothelial and renal cancers | AML blasts (AML-1~64) from human patients  Leukemic mice | zDHHC21-mediated S-acylation of mitochondrial adenylate kinase 2 (AK2) activates OXPHOS and promotes stemness potential, proliferation and | ^33^ |  |
| *zDHHC22* | PM | Gene product is not prognostic | ER negative BrCa cell lines,  ER negative human specimens  Xenograft mouse model | zDHHC22 promotes the proliferation capability *in vitro* and *in vivo*, *via* mTOR - AKT signalling pathway. | ^34^ |  |
| *zDHHC23* | ER, Nucleoplasm | **↑** renal cancer  **↑**endometrial and thyroid cancers | - | - |  |  |
| *zDHHC24* | ER | **↑** glioma | - | - |  |  |
| *LYPLA1* | Nucleoplasm, cytosol | **↑** pancreatic cancer | NSCLC SPC-A-1 and A549 cell lines | LYPLA1 promotes proliferation, migration and mesenchymal markers of NSCLC cellls | ^35^ |  |
| *LYPLA2* |  | **↑** liver cancer  **↑** renal cancer | - | - |  |  |
| *PPT1* | Lysosomes, Golgi | **↑** liver cancer | Melanoma cell line A375P and colorectal HT-29 cancer cell lines  Xenograft mouse model | PPT1 promotes the proliferation *in vitro* and tumour growth *in vivo* | ^36^ | |
|  |  |  | Hepatocellular HCC cell lines Hep 3B and Hep 1-6 (sensitive or resistant to sorafenib)  Animal models of subcutaneous injection of sorafenib-resistant Hep 1-6 | PPT1 promotes cell viability and autophagy flux and reduces tumour volumes *in vivo* | ^37^ | |
| *PPT2* | Golgi | **↑**endometrial cancer | ccRCC A498 and CAKi-1 cell lines | Overexpression of PPT2 represses the proliferation, migration and invasion of ccRCC cells *in vitro*. By reducing epithelial-to-mesenchymal transition (EMT). | ^38^ |  |
| *ABHD10* | Mitochondria | **↑**renal and cervical cancers | - | - |  |  |
| *ABHD17A* | Cytoplasm | **↑**pancreatic, endometrial and head and neck cancers | - | ABHD17A-mediated de-acylation of N-Ras at the PM disrupts N-RAS subcellular localization and attenuates oncogenic N-RAS signalling. | ^39^ |  |
| *ABHD17B* | Cytoplasm | **↑**renal cancer | - | - |  |  |
| *ABHD17C* | Cytoplasm | Gene product is not prognostic | Human PDAC PANC-1 cell line and mouse KPC cell line  and KPC-ABHD17C-OE mice | Upregulation of ABHD17C significantly increases tumour growth *in vivo*  and accelerates the formation of an immunosuppressive microenvironment by promoting glycolysis levels, glycolytic storage capacity glucose uptake ability, lactate secretion, and a significant decrease in the pH value of the microenvironment. | ^40^ |  |

**Table 2: S-acylation of Ca^2+^ Transport proteins**

| **Ca^2+^ transport protein associated to cancer** | | **S-Acylation status** | **Cysteine (s)** | **Enzyme (s)** | **Functional consequence** | **Reference (s)** |
| --- | --- | --- | --- | --- | --- | --- |
| **VGCC** | **CaV1.2** | Yes: α1C subunit  Yes: β2a subunit | C136, C519 and C543  C3 and C4 | - | Increased Ca^2+^ channel activity  Increased Ca^2+^ channel activity by tethering α1C in the PM | ^41,42^ |
|  | **CaV1.3** | Predicted | 4 Cysteines: C488, C1031, C1221 and C1451 | - | - | SwissPalm |
|  | **CaV1.4** | Predicted | 3 Cysteines: C508, C1016 and C1428 | - | - | SwissPalm |
|  | **CaV2.1** | Predicted | 1 cysteine: C1764 | - | - | SwissPalm |
|  | **CaV2.2** | Predicted | 1 cysteine: C1664 | - | - | SwissPalm |
|  | **CaV3.1** | Predicted | 4 cysteines: C1247, C1248, C1419 and C1420 | - | - | SwissPalm |
|  | **CaV3.2** | Predicted | 2 cysteines: C1437 and C1438 | - | - | SwissPalm |
|  | **CaV3.3** | Predicted | 5 cysteines: C72, C555, C556, C1313 and C1314 | - | - | SwissPalm |
| **TRP** | **TRPA1** | Predicted | 2 cysteines: C258 and C834 | - | - | SwissPalm |
|  | **TRPC1** | Yes | Cysteine (s) are not identified  Predicted: 3 cysteines: C703, C736, C737 | zDHHC3 and zDHHC7 | - | ^43^  SwissPalm |
|  | **TRPC3** | Predicted | 1 cysteine: C717 | - | - | SwissPalm |
|  | **TRPC4** | Predicted | 1 cysteine: C678 | - | - | SwissPalm |
|  | **TRPC5** | Yes | C181 | - | Increased channel activity by enhancing ER to PM traffic | ^44^ |
|  | **TRPC6** | Not predicted | - | - | - | SwissPalm |
|  | **TRPC7** | Predicted | 2 cysteines: C728 and C733 | - | - | SwissPalm |
|  | **TRPM1** | Predicted | 1 cysteine: C1128 | - | - | SwissPalm |
|  | **TRPM2** | Predicted | 1 cysteine: C811 | - | - | SwissPalm |
|  | **TRPM3** | Predicted | 7 cysteines: C87, C88, C89, C698, C1187, C1188 and C1606 | - | - | SwissPalm |
|  | **TRPM4** | Predicted | 3 cysteines: C515, C758, C1093 |  |  | SwissPalm |
|  | **TRPM5** | Predicted | 1 cysteine: C378 | - | - | SwissPalm |
|  | **TRPM6** | Yes | C1125 and C1126 | - | - | ^45^ |
|  | **TRPM7** | Yes | C1143, C1144 and C1146 | zDHHC17 in Golgi and ZDHCC5 at PM | Increased channel activity by enhancing Golgi to PM traffic | ^45^ |
|  | **TRPM8** | Yes | Cysteine (s) are not identified  Predicted: 7 cysteines: C68, C1028, C1031, C1032, C1033, C1043, C1044 | zDHHC3 and zDHHC7 | - | ^43^  SwissPalm |
|  | **TRPV1** | Not predicted | - | - | - | SwissPalm |
|  | **TRPV2** | Not predicted | - | - | - | SwissPalm |
|  | **TRPV3** | Predicted | 1 cysteine: C496 | - | - | SwissPalm |
|  | **TRPV4** | Predicted | 2 cysteines: C639 and C645 | - | - | SwissPalm |
|  | **TRPV5** | Predicted | 2 cysteines: C346 and C347 | - | - | SwissPalm |
|  | **TRPV6** | Predicted | 3 cysteines: C110, C386 and C387 | - | - | SwissPalm |
|  | **TRPML1** | Yes | C565, C566 and C567 | - | Dynamic trafficking to the cell surface and increased efficiency of endocytosis | ^46^ |
|  | **TRPML2** | Predicted | 2 cysteines: C551 and C552 | - | - | SwissPalm |
|  | **TRPML3** | Yes | C550, C551 and C549 | zDHHC1 and zDHHC11 | Dynamic trafficking and cellular function | ^47^ |
|  | **TRPP1 (PKD1)** | Predicted | 1 cysteine: C3283 | - | - | SwissPalm |
|  | **TRPP2 (PKD2)** | Not predicted | - | - | - | SwissPalm |
|  | **TRPP3 (PKD2L2)** | Yes | C38 | - | Increased channel activity by enhancing PM tethering | ^48^ |
| **CRAC** | **Orai1** | Yes | C143 | zDHHC3, zDHHC7 and zDHHC20 | Increased Ca^2+^ channel activity by targeting Orai1 to lipid rafts and immune synapses | ^49^ |
|  | **Orai2** | Predicted | 1 cysteine: C16 | - | - | SwissPalm |
|  | **Orai3** | Not predicted | - | - | - | SwissPalm |
|  | **STIM1** | Yes | C437 | zDHHC20 | Increased colocalization with Orai1 and Ca^2+^ entry | ^50^ |
|  | **STIM2** | Predicted | 2 cysteines: C10 and C21 | - | - | SwissPalm |
| **Plasma membrane ATP dependent pumps** | **PMCA1** | Not predicted | - | - | - | SwissPalm |
|  | **PMCA2** | Predicted | 2 cysteines: C635 and C636 | - | - | SwissPalm |
|  | **PMCA4** | Not predicted | - | - | - | SwissPalm |
| **Plasma membrane Ca^2+^ exchanger** | **NCX1** | Yes | C739 | zDHHC5  APT-1 | Decreased exchanger activity by increasing endogenous inhibitory interactions | ^51^ |
| **SR/ER ATP dependent pumps** | **SERCA** | Yes | C12, C344, C349, C364, C471, C636, C674 and C675 | - | - | ^52^ |
| **Mitochondrial Ca^2+^ Uniporter** | **MCU** | Not predicted | - | - | - | SwissPalm |
| **Mitochondrial Ca^2+^ exchangers** | **NCLX1** | Predicted | 1 cysteine: C16 | - | - | SwissPalm |
| **Ca^2+^ release channels** | **IP3 Receptor** | Yes | C56 and C849 (potentially (C2214) | zDHHC6 | Increased IP3R protein levels and Ca^2+^ release activity | ^53^ |
|  | **Ryanodine receptor** | Yes | C24, C36, C253, C305, C537, C1040, C1674, C2021, C2237, C2326, C2363, C2555, C2565, C3170, C3193, C3402 and C3635 | - | Increased RYR1 Ca^2+^ release activity | ^52^ |

**Bibliography**

1. Le X, Mu J, Peng W, et al. DNA methylation downregulated ZDHHC1 suppresses tumor growth by altering cellular metabolism and inducing oxidative/ER stress-mediated apoptosis and pyroptosis. *Theranostics*. 2020;10(21):9495-9511. doi:10.7150/thno.45631

2. Luo CG, Gui CP, Huang GW, et al. Identification of ZDHHC1 as a Pyroptosis Inducer and Potential Target in the Establishment of Pyroptosis-Related Signature in Localized Prostate Cancer. Altieri F, ed. *Oxidative Medicine and Cellular Longevity*. 2022;2022:1-25. doi:10.1155/2022/5925817

3. Peng C, Zhang Z, Wu J, et al. A Critical Role for ZDHHC2 in Metastasis and Recurrence in Human Hepatocellular Carcinoma. *BioMed Research International*. 2014;2014:1-9. doi:10.1155/2014/832712

4. Yan SM, Tang JJ, Huang CY, et al. Reduced Expression of ZDHHC2 Is Associated with Lymph Node Metastasis and Poor Prognosis in Gastric Adenocarcinoma. Suzuki H, ed. *PLoS ONE*. 2013;8(2):e56366. doi:10.1371/journal.pone.0056366

5. Sun Y, Zhu L, Liu P, Zhang H, Guo F, Jin X. ZDHHC2-Mediated AGK Palmitoylation Activates AKT–mTOR Signaling to Reduce Sunitinib Sensitivity in Renal Cell Carcinoma. *Cancer Research*. 2023;83(12):2034-2051. doi:10.1158/0008-5472.CAN-22-3105

6. Sharma C, Rabinovitz I, Hemler ME. Palmitoylation by DHHC3 is critical for the function, expression, and stability of integrin α6β4. *Cell Mol Life Sci*. 2012;69(13):2233-2244. doi:10.1007/s00018-012-0924-6

7. Yao H, Lan J, Li C, et al. Inhibiting PD-L1 palmitoylation enhances T-cell immune responses against tumours. *Nat Biomed Eng*. 2019;3(4):306-317. doi:10.1038/s41551-019-0375-6

8. Zhang X, Hou J, Zhou G, Wang H, Wu Z. zDHHC3-mediated S-palmitoylation of SLC9A2 regulates apoptosis in kidney clear cell carcinoma. *J Cancer Res Clin Oncol*. 2024;150(4):194. doi:10.1007/s00432-024-05737-y

9. Sharma C, Wang HX, Li Q, et al. Protein Acyltransferase DHHC3 Regulates Breast Tumor Growth, Oxidative Stress, and Senescence. *Cancer Research*. 2017;77(24):6880-6890. doi:10.1158/0008-5472.CAN-17-1536

10. Zhao C, Yu H, Fan X, et al. GSK3β palmitoylation mediated by ZDHHC4 promotes tumorigenicity of glioblastoma stem cells in temozolomide-resistant glioblastoma through the EZH2–STAT3 axis. *Oncogenesis*. 2022;11(1):28. doi:10.1038/s41389-022-00402-w

11. Chen X, Ma H, Wang Z, Zhang S, Yang H, Fang Z. EZH2 Palmitoylation Mediated by ZDHHC5 in p53-Mutant Glioma Drives Malignant Development and Progression. *Cancer Research*. 2017;77(18):4998-5010. doi:10.1158/0008-5472.CAN-17-1139

12. Wang Y, Shen N, Yang Y, et al. ZDHHC5-mediated S-palmitoylation of FAK promotes its membrane localization and epithelial-mesenchymal transition in glioma. *Cell Commun Signal*. 2024;22(1):46. doi:10.1186/s12964-023-01366-z

13. Tian H, Lu JY, Shao C, et al. Systematic siRNA Screen Unmasks NSCLC Growth Dependence by Palmitoyltransferase DHHC5. *Molecular Cancer Research*. 2015;13(4):784-794. doi:10.1158/1541-7786.MCR-14-0608

14. Qiu N, Abegg D, Guidi M, Gilmore K, Seeberger PH, Adibekian A. Artemisinin inhibits NRas palmitoylation by targeting the protein acyltransferase ZDHHC6. *Cell Chemical Biology*. 2022;29(3):530-537.e7. doi:10.1016/j.chembiol.2021.07.012

15. Lin Z, Agarwal S, Tan S, et al. Palmitoyl acyltransferase ZDHHC7 inhibits androgen receptor and suppresses prostate cancer. *Oncogene*. 2023;42(26):2126-2138. doi:10.1038/s41388-023-02718-2

16. Jiang Y, Xu Y, Zhu C, et al. STAT3 palmitoylation initiates a positive feedback loop that promotes the malignancy of hepatocellular carcinoma cells in mice. *Sci Signal*. 2023;16(814):eadd2282. doi:10.1126/scisignal.add2282

17. Sudo H, Tsuji AB, Sugyo A, Ogawa Y, Sagara M, Saga T. ZDHHC8 knockdown enhances radiosensitivity and suppresses tumor growth in a mesothelioma mouse model. *Cancer Science*. 2012;103(2):203-209. doi:10.1111/j.1349-7006.2011.02126.x

18. Wang Z, Wang Y, Shen N, et al. AMPKα1-mediated ZDHHC8 phosphorylation promotes the palmitoylation of SLC7A11 to facilitate ferroptosis resistance in glioblastoma. *Cancer Letters*. 2024;584:216619. doi:10.1016/j.canlet.2024.216619

19. Chong X, Zhu L, Yu D, et al. ZDHHC9 promotes colon tumor growth by inhibiting effector T cells. *Oncol Lett*. 2022;25(1):5. doi:10.3892/ol.2022.13591

20. Lin Z, Huang K, Guo H, et al. Targeting ZDHHC9 potentiates anti-programmed death-ligand 1 immunotherapy of pancreatic cancer by modifying the tumor microenvironment. *Biomedicine & Pharmacotherapy*. 2023;161:114567. doi:10.1016/j.biopha.2023.114567

21. Liu Y, Zhao X, Seitz A, et al. Circular ZDHHC11 supports Burkitt lymphoma growth independent of its miR-150 binding capacity. *Sci Rep*. 2024;14(1):8730. doi:10.1038/s41598-024-59443-3

22. Zhang X, Liao X, Wang M, et al. Inhibition of palmitoyltransferase ZDHHC12 sensitizes ovarian cancer cells to cisplatin through ROS ‐mediated mechanisms. *Cancer Science*. 2024;115(4):1170-1183. doi:10.1111/cas.16085

23. Yuan M, Chen X, Sun Y, et al. ZDHHC12-mediated claudin-3 S-palmitoylation determines ovarian cancer progression. *Acta Pharmaceutica Sinica B*. 2020;10(8):1426-1439. doi:10.1016/j.apsb.2020.03.008

24. Perez CJ, Mecklenburg L, Jaubert J, et al. Increased Susceptibility to Skin Carcinogenesis Associated with a Spontaneous Mouse Mutation in the Palmitoyl Transferase Zdhhc13 Gene. *Journal of Investigative Dermatology*. 2015;135(12):3133-3143. doi:10.1038/jid.2015.314

25. Sun Y, Li X, Yin C, et al. AMPK Phosphorylates ZDHHC13 to Increase MC1R Activity and Suppress Melanomagenesis. *Cancer Research*. 2023;83(7):1062-1073. doi:10.1158/0008-5472.CAN-22-2595

26. Yeste‐Velasco M, Mao X, Grose R, et al. Identification of  *ZDHHC14*  as a novel human tumour suppressor gene. *The Journal of Pathology*. 2014;232(5):566-577. doi:10.1002/path.4327

27. Liu ZY, Lan T, Tang F, et al. ZDHHC15 promotes glioma malignancy and acts as a novel prognostic biomarker for patients with glioma. *BMC Cancer*. 2023;23(1):420. doi:10.1186/s12885-023-10883-6

28. Zeyu Liu, Chuanqing Jing, Wei Zhang. METTL3-mediated m6A modification enhances ZDHHC16 expression in nonsmall-cell lung cancer patients, attenuating ferroptosis by suppressing CREBubiquitination. *Cell Mol Biol (Noisy-le-grand)*. 2024;70(2):30-37. doi:10.14715/cmb/2024.70.2.5

29. Chen X, Hao A, Li X, et al. Activation of JNK and p38 MAPK Mediated by ZDHHC17 Drives Glioblastoma Multiforme Development and Malignant Progression. *Theranostics*. 2020;10(3):998-1015. doi:10.7150/thno.40076

30. Pei X, Li KY, Shen Y, et al. Palmitoylation of MDH2 by ZDHHC18 activates mitochondrial respiration and accelerates ovarian cancer growth. *Sci China Life Sci*. 2022;65(10):2017-2030. doi:10.1007/s11427-021-2048-2

31. Liang S, Zhang X, Li J. Zinc finger Asp-His-His-Cys palmitoyl -acyltransferase 19 accelerates tumor progression through wnt/β-catenin pathway and is upregulated by miR-940 in osteosarcoma. *Bioengineered*. 2022;13(3):7367-7379. doi:10.1080/21655979.2022.2040827

32. Tomić G, Sheridan C, Refermat AY, et al. Palmitoyl transferase ZDHHC20 promotes pancreatic cancer metastasis. *Cell Reports*. 2024;43(5). doi:10.1016/j.celrep.2024.114224

33. Shao X, Xu A, Du W, et al. Palmitoyltransferase ZDHHC21 regulates oxidative phosphorylation to induce differentiation block and stemness in AML. *Blood Journal*. Published online May 22, 2023:blood.2022019056. doi:10.1182/blood.2022019056

34. Huang J, Li J, Tang J, et al. ZDHHC22-mediated mTOR palmitoylation restrains breast cancer growth and endocrine therapy resistance. *Int J Biol Sci*. 2022;18(7):2833-2850. doi:10.7150/ijbs.70544

35. Mohammed A, Zhang C, Zhang S, et al. Inhibition of cell proliferation and migration in non‑small cell lung cancer cells through the suppression of LYPLA1. *Oncol Rep*. Published online November 9, 2018. doi:10.3892/or.2018.6857

36. Rebecca VW, Nicastri MC, Fennelly C, et al. PPT1 Promotes Tumor Growth and Is the Molecular Target of Chloroquine Derivatives in Cancer. *Cancer Discovery*. 2019;9(2):220-229. doi:10.1158/2159-8290.CD-18-0706

37. Xu J, Su Z, Cheng X, et al. High PPT1 expression predicts poor clinical outcome and PPT1 inhibitor DC661 enhances sorafenib sensitivity in hepatocellular carcinoma. *Cancer Cell Int*. 2022;22(1):115. doi:10.1186/s12935-022-02508-y

38. Yuan C, Xiong Z, Shi J, et al. Overexpression of PPT2 Represses the Clear Cell Renal Cell Carcinoma Progression by Reducing Epithelial-to-mesenchymal Transition. *J Cancer*. 2020;11(5):1151-1161. doi:10.7150/jca.36477

39. Lin DTS, Conibear E. ABHD17 proteins are novel protein depalmitoylases that regulate N-Ras palmitate turnover and subcellular localization. *eLife*. 2015;4:e11306. doi:10.7554/eLife.11306

40. Zhang W, Xie Y, Yu X, et al. ABHD17C, a metabolic and immune-related gene signature, predicts prognosis and anti-PD1 therapy response in pancreatic cancer. *Discov Onc*. 2023;14(1):87. doi:10.1007/s12672-023-00690-7

41. Kuo CWS, Dobi S, Gök C, et al. Palmitoylation of the pore-forming subunit of Ca(v)1.2 controls channel voltage sensitivity and calcium transients in cardiac myocytes. *Proc Natl Acad Sci USA*. 2023;120(7):e2207887120. doi:10.1073/pnas.2207887120

42. Kazim AS, Storm P, Zhang E, Renström E. Palmitoylation of Ca2+ channel subunit CaVβ2a induces pancreatic beta-cell toxicity via Ca2+ overload. *Biochemical and Biophysical Research Communications*. 2017;491(3):740-746. doi:10.1016/j.bbrc.2017.07.117

43. Oku S, Takahashi N, Fukata Y, Fukata M. In Silico Screening for Palmitoyl Substrates Reveals a Role for DHHC1/3/10 (zDHHC1/3/11)-mediated Neurochondrin Palmitoylation in Its Targeting to Rab5-positive Endosomes. *Journal of Biological Chemistry*. 2013;288(27):19816-19829. doi:10.1074/jbc.M112.431676

44. Hong C, Choi SH, Kwak M, et al. TRPC5 channel instability induced by depalmitoylation protects striatal neurons against oxidative stress in Huntington’s disease. *Biochimica et Biophysica Acta (BBA) - Molecular Cell Research*. 2020;1867(2):118620. doi:10.1016/j.bbamcr.2019.118620

45. Gao X, Kuo CW, Main A, et al. Palmitoylation regulates cellular distribution of and transmembrane Ca flux through TrpM7. *Cell Calcium*. 2022;106:102639. doi:10.1016/j.ceca.2022.102639

46. Vergarajauregui S, Puertollano R. Two Di‐Leucine Motifs Regulate Trafficking of Mucolipin‐1 to Lysosomes. *Traffic*. 2006;7(3):337-353. doi:10.1111/j.1600-0854.2006.00387.x

47. Kim SW, Kim DH, Park KS, et al. Palmitoylation controls trafficking of the intracellular Ca ^2+^ channel MCOLN3/TRPML3 to regulate autophagy. *Autophagy*. 2019;15(2):327-340. doi:10.1080/15548627.2018.1518671

48. Zheng W, Yang J, Beauchamp E, et al. Regulation of TRPP3 Channel Function by N-terminal Domain Palmitoylation and Phosphorylation. *Journal of Biological Chemistry*. 2016;291(49):25678-25691. doi:10.1074/jbc.M116.756544

49. Carreras-Sureda A, Abrami L, Ji-Hee K, et al. S-acylation by ZDHHC20 targets ORAI1 channels to lipid rafts for efficient Ca2+ signaling by Jurkat T cell receptors at the immune synapse. *eLife*. 2021;10:e72051. doi:10.7554/eLife.72051

50. Kodakandla G, West SJ, Wang Q, et al. Dynamic S-acylation of the ER-resident protein stromal interaction molecule 1 (STIM1) is required for store-operated Ca2+ entry. *Journal of Biological Chemistry*. 2022;298(9):102303. doi:10.1016/j.jbc.2022.102303

51. Gök C, Plain F, Robertson AD, et al. Dynamic Palmitoylation of the Sodium-Calcium Exchanger Modulates Its Structure, Affinity for Lipid-Ordered Domains, and Inhibition by XIP. *Cell Reports*. 2020;31(10):107697. doi:10.1016/j.celrep.2020.107697

52. Chaube R, Hess DT, Wang YJ, et al. Regulation of the Skeletal Muscle Ryanodine Receptor/Ca2+-release Channel RyR1 by S-Palmitoylation. *Journal of Biological Chemistry*. 2014;289(12):8612-8619. doi:10.1074/jbc.M114.548925

53. Fredericks GJ, Hoffmann FW, Rose AH, et al. Stable expression and function of the inositol 1,4,5-triphosphate receptor requires palmitoylation by a DHHC6/selenoprotein K complex. *Proc Natl Acad Sci USA*. 2014;111(46):16478-16483. doi:10.1073/pnas.1417176111
